# Biphasic insulin Aspart 30 vs. NPH plus regular human insulin in type 2 diabetes patients; a cost-effectiveness study

**DOI:** 10.1186/s12902-016-0116-8

**Published:** 2016-06-09

**Authors:** Amir Farshchi, Rokhsareh Aghili, Maryam Oskuee, Marjan Rashed, Sina Noshad, Abbas Kebriaeezadeh, Maryam Kia, Alireza Esteghamati

**Affiliations:** Department of Pharmacoeconomics and Pharmaceutical Administration, School of Pharmacy, Tehran University of Medical Sciences, Tehran, Iran; Endocrine Research Center, Institute of Endocrinology and Metabolism, Iran University of Medical Sciences, Tehran, Iran; Pharmaceutical Sciences branch, Islamic Azad University, Tehran, Iran; Endocrinology and Metabolism Research Center (EMRC), Vali-Asr Hospital, School of Medicine, Tehran University of Medical Sciences, P.O. Box 13145-784, Tehran, Iran; Department of Internal Medicine, Dr. Ziaeian Hospital, Tehran University of Medical Sciences, Tehran, Iran

**Keywords:** Type 2 diabetes mellitus, Insulin, Biphasic insulin aspart 30, Cost, Cost-effectiveness and QALY

## Abstract

**Background:**

The aim of this study was to compare the efficacy, safety, costs, and cost-effectiveness of biphasic insulin aspart 30 (BIAsp 30) with NPH plus regular human insulin (NPH/Reg) in patients with type 2 diabetes mellitus (T2DM).

**Methods:**

It was a Single-center, parallel-group, randomized, clinical trial (Trial Registration: NCT01889095). One hundred and seventy four T2DM patients with poorly controlled diabetes (HbA1c ≥ 8 % (63.9 mmol/mol)) were randomly assigned to trial arms (BIAsp 30 and NPH/Reg) and were followed up for 48 weeks. BIAsp 30 was started at an initial dose of 0.2–0.6 IU/Kg in two divided doses and was titrated according to the glycemic status of the patient. Similarly, NPH/Reg insulin was initiated at a dose of 0.2–0.6 IU/Kg with a 2:1 ratio and was subsequently titrated. Level of glycemic control, hypoglycemic events, direct and indirect costs, quality adjusted life year (QALY) and incremental cost-effectiveness ratio have been assessed.

**Results:**

HbA1c, Fasting plasma glucose (FPG), and two-hour post-prandial glucose (PPG) were improved in both groups during the study (*P* < 0.05 for all analyses). Lower frequencies of minor, major, and nocturnal hypoglycemic episodes were observed with BIAsp 30 (*P* < 0.05). Additionally, BIAsp 30 was associated with less weight gain and also higher QALYs (*P* < 0.05). Total medical and non-medical costs were significantly lower with BIAsp 30 as compared with NPH/Reg (930.55 ± 81.43 USD vs. 1101.24 ± 165.49 USD, *P* = 0.004). Moreover, BIAsp 30 showed lower ICER as a dominant alternative.

**Conclusions:**

Despite being more expensive, BIAsp 30 offers the same glycemic control as to NPH/Reg dose-dependently and also appears to cause fewer hypoglycemic events and to be more cost-effective in Iranian patients with type 2 diabetes.

## Background

Type 2 diabetes mellitus (T2DM) is the most common metabolic disease in the world with highly cost-demanding complications [1, 2]. Glycemic control is the mainstay of treatment in patients with diabetes mellitus and can be achieved via medical nutritional therapy [3, 4], physical activity, oral medications, and insulin therapy [5]. Tight glycemic control, with glucose concentrations as close as possible to the non-diabetic range, has been demonstrated to reduce diabetes-related complications including retinopathy, nephropathy, cardiovascular diseases and overall mortality [6, 7]. However, incident hypoglycemia poses a significant barrier to achievement of the targeted level of glycemic control; even a single episode could result in catastrophic consequences [8]. American Diabetes Association (ADA) recommends that, irrespective of the treatment strategy employed, precautions should be implemented in order to avoid hypoglycemia [5]. Given the lifelong course of diabetes, treatment strategies should take a number of aspects into consideration, among which medication efficacy, patient satisfaction, and costs of therapy are of particular importance [9]. It is recommended that if lifestyle modification and full tolerated doses of one or two oral glucose lowering drugs (OGLDs) fail to achieve or sustain glycemic goals, then insulin therapy should be initiated [10]. Over the past decade, insulin analogs have gained recognition since they offer numerous advantages over the traditional preparations with regard to blood glucose variability, number of injections needed, patient satisfaction, and life expectancy [11–14].

Despite these advantages, the cost of insulin analogs is a major problem. For instance, Palmer and colleagues have shown that switching from traditional preparations to biphasic insulin Aspart 30 (BIAsp 30) would result in an additional $ 9000 in terms of life-time direct medical costs [15]. A number of previous studies have delineated the efficacy, safety, and cost-effectiveness of BIAsp 30 in patients with diabetes [14–17]; yet the cost-effectiveness of such therapy has not been explored in Iran. The aim of the present piggyback study was to investigate the cost-effectiveness of BIAsp 30 using the data from a clinical trial Iranian patients with T2DM.

## Methods

### Study design and participants

The analysis of the present study is based on the data collected in the single-center, randomized, parallel-group, clinical trial conducted between July 2011 and October 2012 in Diabetes clinic of the Vali-Asr hospital (a teaching hospital affiliated with Tehran University of Medical Sciences, Tehran, Iran). Two hundred and four T2DM patients currently taking OGLDs were initially assessed. Patients were included if had HbA1c >8.0 % despite adequate therapy with lifestyle modification and one or two classes of OGLDs. Exclusion criteria were as follows: (1) recent surgery or clinically significant infection, (2) treatment with glucocorticoids, (3) incidence of severe hypoglycemic episode requiring hospital admission or visit by a healthcare professional; (4) previous use of any type of insulin; (5) presence of diabetes retinopathy significant enough to require treatment in the past 6 months; (6) estimated glomerular filtration rate < 50 mL/min/1.73 m^2^; (7) pregnancy, breast-feeding, planning to become pregnant in the next year, or use of inadequate contraceptive measuring in women of child-bearing age; and (8) current participation in other clinical studies. A total of 174 patients met the inclusion criteria and were assigned to either of the trial arms with the aid of randomization software.

### Ethics, consent and permissions

The trial was approved by the Institutional Review Board at the Tehran University of Medical Sciences (project number: 90-03-33-15600) and it is also registered with ClinicalTrial.gov (Reg. No.: NCT01889095). After fully disclosing the purpose of the study, written informed consent was obtained from each patient. All procedures involving human subjects were conducted in accordance with the guidelines laid down the recent revision of Helsinki declaration.

### Interventions

The insulin therapies were prescribed by a single physician in the clinic (A.E.). The starting dose of BIAsp 30 (NovoMix^®^ 30-pen, NovoNordisk) was 0.2–0.6 unit/kg per day injected in two divided doses (pre-breakfast and pre-dinner). The other arm of the trial received NPH/Reg insulin (Exir pharmaceuticals, Lorestan, Iran) in a Ratio of 2:1 with initiation dose of 0.2–0.6 unit/kg in injected in two divided doses. Two-thirds of the dose was given before breakfast and the remainder before dinner. Initiation of NPH/Reg therapy was provided in an in-patient setting for careful monitoring of blood glucose. Continuation and/or dose modification of OGLDs at the time of starting the insulin regimen and throughout the study was entirely at the discretion of the treating physician guided by glycemic control achieved. Patients were asked to record their 7-point blood glucose values in three consecutive days before each visit. Seven-point self-monitoring blood glucose includes three pre-meals, three post-meals, and one bedtime blood glucose readings, each day. Patients received instruction from a nurse regarding usage of the glucometer for capillary glucose monitoring. Insulin doses were adjusted by a titration regimen according to the self-monitored blood glucose sheets. For both groups, treatment goals were set as follows: fasting blood glucose of 80–120 mg/dl, postprandial glucose <160 mg/dl, HbA1c <7 % and the before dinner blood glucose target for the NPH/Reg insulin group was 100 mg/dl. Stepwise increases in dosage of insulin in both arms was done depending on the pre-meal blood glucose values to achieve targets for plasma glucose (PG) as follows: +2 IU/day where 126 mg/dl < PG ≤ 140 mg/dl, +4 IU/day where 140 mg/dl < PG ≤ 160 mg/dl, +6 IU/day where 160 mg/dl < PG ≤ 180 mg/dl, +8 IU/day where 180 mg/dl < PG ≤ 200 mg/dl and +10 IU/day where PG > 200 mg/dl [18, 19], unless hypoglycemia occurred. Hypoglycemia was defined as blood glucose <70 mg/dl [20]. Severe hypoglycemia was defined as an event with symptoms consistent with hypoglycemia where the individual required the assistance of another person and was not treated with oral carbohydrate due to confusion or being unconsciousness and was associated with a blood glucose level <40 mg/dl with recovery with intravenous glucose, or glucagon administration. Nocturnal hypoglycemia was defined as hypoglycemia occurred at night and is commonly known as hypoglycemia while asleep. Data were collected at each visit during the study period (see below).

### Assessments

Patients in the both arms of the trial were visited at baseline, and then with 12-weeks interval thereafter. Therefore, five visits at weeks 0, 12, 24, 36, and 48 were conducted. The initial visit included evaluation for meeting the inclusion criteria followed by acquiring a detailed medical history along with performance of a thorough physical examination. Specific checklists for determination of medical, non-medical and productivity costs were also completed at each visit during study period (between July 2011 and October 2012). QALY was assessed using the self-administered standard EQ-5D-3 L questionnaire [21].

### Laboratory measurements

Laboratory evaluations were performed at baseline and every 12 weeks thereafter. After an overnight fasting of at least 12 h, venous blood samples were drawn and were sent to hospital laboratory for analysis. Fasting plasma glucose (FPG) concentrations were determined using the glucose oxidase method. Serum concentrations of two-hour post-prandial glucose (PPG) were assessed using a glucose analyzer (YSI 2700 Select, YSI, Inc., Yellow Springs, OH). Percentage of glycated hemoglobin A1c (HbA1c) was measured using the high performance liquid chromatography (HPLC) method [22]. Serum concentrations of total cholesterol, high-density lipoprotein cholesterol (HDL), low density lipoprotein cholesterol (LDL), and triglycerides were determined using enzymatic methods with available commercial kits (Pars Azmun, Karaj, Iran) in a Hitachi 704 automatic analyzer (Tokyo, Japan) [23].

### Analysis of costs

Direct medical/non-medical costs and indirect costs as described and published by Farshchi et al. [2], were calculated using a checklist. Costs from the societal perspective were converted from Iranian Rials (IRR) into USA dollar (USD) at an official exchange rate of 12,260 IRR/1USD 2012 to have an international comparison [24].

### Utility calculation

Utility scores were calculated by two well-known measurements; European Quality of Life-5 Dimensions,-3 Levels (EQ-5D-3 L) from 0 to 1 and Visual Analogue Scale (VAS) between 0 and 100. These are two standardized measures of health status and quality adjusted life year (QALY), developed by the EuroQol group in order to provide a simple, generic measure of health for clinical and economic appraisal [25].

### Cost-effectiveness analysis

QALYs and number of reduction in hypoglycemic events were considered as outcomes and the incremental cost effectiveness ratio (ICER) per patient was calculated according to the below formula: ICER = ∆Cost/ ∆Outcome [26, 27]. The comparison of ICERs per each outcome was held afterwards. Based on recommendation of the World Health Organization (WHO) [28], the ICER were compared with the gross domestic product (GDP) per capita. The GDP per capita of Iran was recorded as 6578 USD in 2012 [29].

### Statistical analysis

Continuous variables are presented as mean ± standard deviation (SD) and categorical ones as proportions. Between groups comparisons were conducted using Student’s *t*-test for continuous variables, and Pearson’s Chi square for categorical ones. Difference in the outcome variables of interest between trial arms was investigated using the analysis of variance (ANOVA) and paired *t*-test methods. All analyses were conducted using Software Package for Social Sciences (version 14 for Windows; SPSS Inc., Chicago, IL). *P* values < 0.05 were considered statistically significant.

## Results

**Fig. 1:**
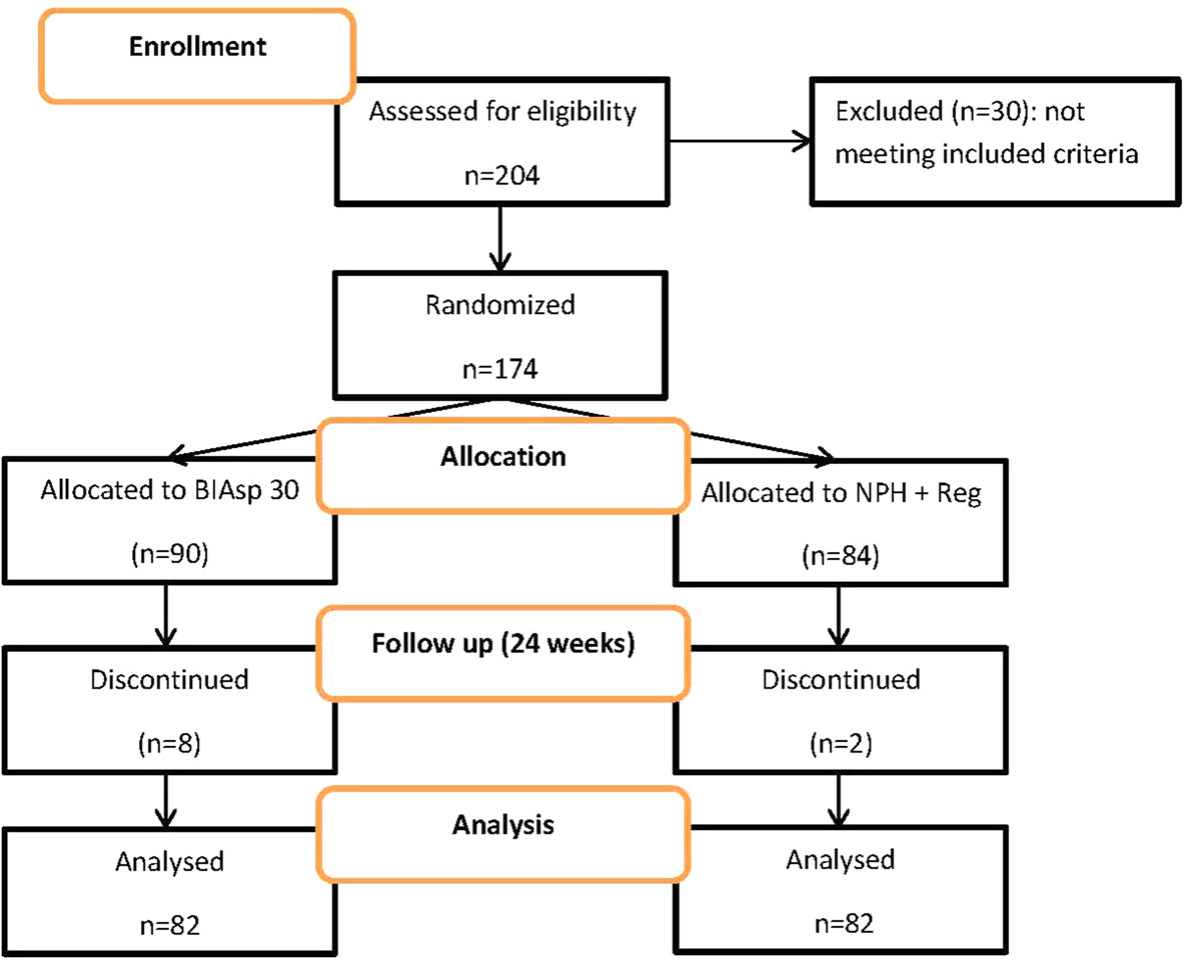
CONSORT flow diagram

Two hundred and four subjects were initially assessed; 174 met the inclusion criteria and were therefore allocated to BIAsp (*n* = 90) or NPH/Reg (*n* = 84) arms of the trial. Eight subjects in the BIAsp 30, and two in the NPH/Reg arm did not return for the follow up visits and were lost to follow up. Overall, 164 patients or 94.3 % completed the trial. The CONSORT flow diagram of the trial is depicted in Fig. 1. Baseline characteristics of trial participants are presented in Table 1. Patients’ characteristics were comparable between the two arms of the trial. Baseline HbA1c concentrations were comparable (9.55 ± 1.03 vs. 9.97 ± 1.52 %, *P* = 0.576) and disease duration was 13.60 ± 3.12 years vs. 15.62 ± 4.86 years in BIAsp 30 and insulin NPH/Reg insulin group (*P* = 0.194), respectively.

**Table 1:** Baseline characteristics of the trial participants

		Trial arms		
		BIAsp 30 (*n* = 82)	NPH/Reg (*n* = 82)	*P* value
Age (years)		58.50 ± 14.04	57.24 ± 10.88	0.072
Sex (male/female)		33/49	29/53	0.343
Diabetes Duration (years)		13.60 ± 3.12	15.62 ± 4.86	0.194
HbA1c (%)		9.55 ± 1.03	9.97 ± 1.52	0.576
TG (mg/dl)		183.19 ± 11.33	191.22 ± 55.11	0.153
Total Chol (mg/dl)		184.41 ± 21.31	176.33 ± 22.30	0.254
LDL (mg/dl)		103.72 ± 19.70	110.39 ± 14.56	0.348
HDL (mg/dl)		45.34 ± 11.05	48.55 ± 15.12	0.176
BMI (kg/m^2^)		29.37 ± 6.78	31.78 ± 7.52	0.211
Prior OGLD treatment (*n*, %)	Metformin	25 (30.48)	27 (32.92)	0.972
	Sulfonylurea	9 (10.97)	9 (10.97)	
	Metformin + Sulfonylurea	40 (48.78)	39 (47.56)	
	Thiazolidinedione	4 (4.87)	5 (6.09)	

Abbreviations: *BIAsp* biphasic insulin aspart 30, *NPH/Reg* NPH plus regular human insulin, *HbA1c* hemoglobin A1c, *TG* triglyceride, *Chol* cholesterol, *LDL* low density lipoprotein, *HDL* high density lipoprotein, *BMI* body mass index, *OGLD* oral glucose lowering drugs

### Insulin therapy

No serious adverse drug reactions were reported. In BIAsp 30, insulin dose increased over the trial course from a daily mean starting dose of 0.32 ± 0.06 to 0.76 ± 0.14 IU/kg. The pre-breakfast dose of BIAsp 30 increased from the mean starting dose of 0.16 ± 0.03 to 0.38 ± 0.07 IU/kg, whereas the pre-dinner dose increased from the mean starting dose of 0.16 ± 0.04 to 0.37 ± 0.05 IU/kg at the end of the trial. Similarly, NPH/Reg insulin dose increased from a daily mean starting dose of 0.34 ± 0.05 to 0.80 ± 0.07 IU/kg. The pre-breakfast dose of NPH/Reg insulin increased from the mean starting dose of 0.18 ± 0.04 to 0.41 ± 0.06 IU/kg, while pre-dinner dose increased from the mean starting dose of 0.18 ± 0.03 to 0.40 ± 0.07 IU/kg by the end of the trial.

### Glycemic control

Table 2 shows metabolic control for each trial arm at baseline and after 48 weeks of insulin therapy. Overall, the percentage of patients achieving HbA1c level of <7.0 % was 49 % at week 48 (65 % for BIAsp 30 and 33 % for NPH/Reg insulin); considerable decrease in HbA1c values were seen with both NPH/Reg insulin and BIAsp 30. Although BIAsp 30 decreased FPG and PPG concentrations to a larger extent, the difference did not reach statistical significance (132.00 ± 13.2 vs. 144.33 ± 8.16, *P* = 0.122). On the other hand HbA1c levels decreased 2.40 ± 1.28 % in BIAsp 30 and 2.34 ± 1.53 % in NPH/Reg insulin groups while there was no statistically significant difference between groups (*P* = 0.233).

**Table 2:** Metabolic control and QALY for the study population at baseline and after 48 weeks of insulin analog therapy

	Trial arms					
	BIAsp 30 (*n* = 82)			NPH/Reg Insulin (*n* = 82)		
	Baseline	48 weeks	*P* value	Baseline	48 weeks	*P* value
HbA1c (%)	9.55 ± 1.03	7.15 ± 0.24	0.021	9.97 ± 1.52	7.62 ± 0.21	0.011
FPG (mg/dl)	208.10 ± 4.51	144.33 ± 8.16	0.029	185.76 ± 12.1	132.00 ± 13.2	0.027
PPG (mg/dl)	276.84 ± 9.2	211.51 ± 10.3	0.032	239.25 ± 12.55	180.88 ± 12.90	0.019
TG (mg/dl)	183.19 ± 11.33	173.44 ± 12.50	0.247	191.22 ± 55.11	170.42 ± 40.18	0.631
Total Chol (mg/dl)	184.41 ± 21.31	169.41 ± 15.13	0.471	176.33 ± 22.30	161.28 ± 18.34	0.259
LDL (mg/dl)	103.72 ± 19.70	101.89 ± 16.42	0.554	110.39 ± 14.56	98.44 ± 19.45	0.131
HDL (mg/dl)	45.34 ± 11.05	49.45 ± 18.65	0.398	48.55 ± 15.12	47.47 ± 19.35	0.228
BMI (kg/m^2^)	29.37 ± 6.78	29.59 ± 3.62	0.421	31.78 ± 7.52	33.88 ± 2.89	0.166
QALY						
EQ-5D-3 L	0.59 ± 0.03	0.73 ± 0.04	0.031	0.64 ± 0.11	0.66 ± 0.06	0.082
VAS	0.61 ± 0.05	0.75 ± 0.06	0.026	0.69 ± 0.04	0.71 ± 0.08	0.074

Abbreviations: *BIAsp* biphasic insulin aspart 30, *NPH/Reg* NPH plus regular human insulin, *HbA1c* hemoglobin A1c, *TG* triglyceride, *Chol* cholesterol, *LDL* low density lipoprotein, *HDL* high density lipoprotein, *BMI* body mass index, *QALY* quality adjusted life year, *EQ-5D-3 L* European quality of life-5 dimensions -3 levels, *VAS* and visual analogue scale

Hypoglycemic events were evaluated as minor, major and nocturnal hypoglycemic episodes and are described in Table 3. Minor, major, and nocturnal events were more frequent among patients in the NPH/Reg arm (*P* < 0.05 in all cases). Consequently, the frequency of total events were higher in NPH/Reg arm (*P* = 0.002).

**Table 3:** Frequency of hypoglycemic events in trial arms reported as per person-year

	Trial arms		
Type of event	BIAsp 30 (*n* = 82)	NPH/Reg (*n* = 82)	*P* value
Minor	7.28 ± 3.51	21.60 ± 7.18	0.012
Major	2.76 ± 1.12	7.68 ± 4.32	0.031
Nocturnal	10.04 ± 3.61	21.48 ± 6.72	0.026
Total	20.08 ± 5.60	50.76 ± 11.50	0.002

Abbreviations: *BIAsp* biphasic insulin aspart 30, *NPH/Reg* NPH plus regular human insulin

BIAsp 30 was also associated with less weight gain (+0.22 ± 1.55 vs. +2.10 ± 2.69, *P* = 0.045).

### Costs

#### Direct costs

Mean direct costs were 595.15 ± 30.15USD for BIAsp 30 and 726.34 ± 60.34 USD for NPH/Reg arm (Table 4). Total direct medical costs in NPH/Reg insulin arm were higher than BIAsp 30 group (*P* = 0.017). Medications and inpatients cost were significantly higher in NPH/Reg insulin group (*P* < 0.05), it was due to more admissions and longer stay in hospital, while there were not statistically significant differences in laboratory and clinical visits between groups (*P* > 0.05). Although direct nonmedical costs in NPH/Reg insulin group were higher than BIAsp 30 group (Table 4), this difference did not reach statistical significance (*P* = 0.332).

**Table 4:** Direct and indirect costs of insulin therapy in trial arms reported in USD

		Trial arms		
Type of cost	Subcategory	BIAsp 30 (*n* = 82)	NPH/Reg Insulin (*n* = 82)	*P* value
Direct costs	Laboratory	55.3 ± 12.9	51.1 ± 11.1	0.732
	Medications	225.8 ± 41.7	60.5 ± 22.6	0.024
	Clinical visits	55.9 ± 13.8	56.2 ± 9.3	0.815
	Inpatient	235.4 ± 51.8	535.9 ± 37.8	0.009
	Non-medical	17.7 ± 4.9	22.0 ± 4.2	0.332
Indirect costs	Productivity loss	340.4 ± 42.2	375.4 ± 70.3	0.271
Total		930.5 ± 81.4	1101.3 ± 165.5	0.004

Abbreviations: *USD* U.S. dollars, *BIAsp* biphasic insulin aspart 30, *NPH/Reg* NPH plus regular human insulin

### Indirect costs

Costs of lost productivity were higher in NPH/Reg insulin group (Table 4). Mean indirect costs for BIAsp 30 was 340.4 ± 42.21 USD while this figure was 375.45 ± 70.33 USD for NPH/Reg arm with no significant difference (*P* = 0.271). Total cost was estimated to be 930.55 ± 81.43 USD for BIAsp 30 and 1101.24 ± 165.49 USD for NPH/Reg arm.

### Utility

EQ-5D-3 L and VAS scores improved during the trial in patients in both groups. But treatment with BIAsp 30 significantly lead to higher QALYs (*P* = 0.011). There was no significant difference in QALY scores during the trial in NPH/Reg group. Mean difference of QALY scores for EQ-5D-3 L were 0.12 ± 0.05 and 0.04 ± 0.02 for BIAsp 30 and NPH/Reg insulin groups after 48 weeks, while there were 0.14 ± 0.06 and 0.05 ± 0.04 for VAS respectively (Table 2).

### Cost effectiveness analysis

The cut-off for ICER was approximately 20000 $ per QALY. Regarding major clinical outcomes (i.e. hypoglycemia events and QALY) BIAsp 30 showed lower ICER as a dominant alternative (Table 5).

**Table 5:** Cost-effectiveness analysis for hypoglycemic events and utility outcomes

	Trial arms	
	BIAsp 30 (*n* = 82)	NPH/Reg (*n* = 82)
Hypoglycemia event per person-year	20.08 ± 5.60	50.76 ± 11.50
Mean difference of QALY (EQ-5D-3 L)	0.12 ± 0.05	0.04 ± 0.02
Mean difference of QALY (VAS)	0.14 ± 0.06	0.05 ± 0.04
Costs (USD)	930.55 ± 81.43	1101.24 ± 165.49
ICER	Dominant	---

Abbreviations: *BIAsp* biphasic insulin aspart 30, *NPH/Reg* NPH plus regular human insulin, *QALY* quality adjusted life year, *EQ-5D-3 L* European quality of life-5 dimensions -3 levels, *VAS* and visual analogue scale, *USD* U.S. dollars, *ICER* incremental cost-effectiveness ratio

## Discussion

In the present randomized clinical trial, efficacy, safety, cost, and cost-effectiveness of BIAsp 30 and NPH/Reg insulin regimens were compared. After 48 weeks, patients in both trial arms experienced a significant improvement in glycemic control as evidenced by substantial decreases in serum concentrations of FPG, PPG, and HbA1c. By the end of the trial, 65 % of patients in the BIAsp 30 arm, and 33 % of the patients in the NPH/Reg arm achieved glycemic goals delineated by the ADA [5] (*P* = 0.032). Although observed rate for BIAsp 30 is higher, it is still significantly lower than the rated reported in clinical trials of shorter or equal duration. Raskin et al., in a trial of insulin-naïve T2DM patients treated with 5–6 units BIAsp 30 twice daily were able to achieve HbA1c < 7.0 % in 66 % of patients by week 28 [30]. In the 1–2–3 study by Garber et al., a step-by-step incremental regimen of BIAsp was able to attain ADA targets in 70 % of T2DM patients by 32 weeks of injections twice daily. This rate increased to 77 % by an additional 16 weeks of injections thrice daily [31]. In a double-blind parallel-group randomized trial of 403 T2DM patients, efficacy and safety of BIAsp 30 with NPH was compared. After 16-weeks of insulin therapy, BIAsp 30 proved to be superior in reducing postprandial glucose and was at least as effective in HbA1c reduction Safety and efficacy profiles of the two regimens were also comparable [32]. Similar results have been replicated in type 1 and type 2 diabetes populations [33]. Along the same lines, McSorely et al. demonstrated that compared with biphasic human insulin 30, BIAsp 30 twice daily provides greater mean insulin concentrations both after breakfast and after dinner; induces earlier postprandial peaks; and finally produces lower glucose excursions four hours after the injection [34].

The most serious adverse effect of insulin therapy is hypoglycemia, but the frequency and severity of this effect is less in type 2 diabetes than in type 1 diabetes [35]. In the United Kingdom Prospective Diabetes Study (UKPDS) major hypoglycemia occurred in 2.3 % of patients per year who were treated with insulin compared with rates of 0.6 % in those on sulfonylurea therapy [36]. The risk of hypoglycemia increases significantly when the HbA1c level is below 7.4 % [37]. Despite comparable glycemic control between BIAsp 30 and NP/Reg, hypoglycemia episodes (including minor, major, and nocturnal events) were significantly more frequent among NPH/Reg patients. This might be due to slow absorption of regular human insulin from the subcutaneous tissue which leads to a delayed peak two to three hours after injection [38]. Consequently, post-meal hyperglycemia followed by delayed postprandial hypoglycemia ensues. Additionally, absorption rates for the conventional basal NPH insulin vary and its duration of action is shorter than 24 h. These pharmacodynamics limitations may result in high fasting blood glucose and nocturnal hypoglycemia [39, 40]. Contrarily, biphasic analogs such as BIAsp 30 have a more rapid onset of action that results in more effective reduction of postprandial glucose, diminishing the chance of occurrence of hypoglycemia [41]. The efficacy and safety of BIAsp 30 have been widely documented in randomized clinical trials and observational studies [17, 31, 42, 43]. The results achieved in this study showed that initiating insulin therapy with BIAsp 30 resulted in lower rates of hypoglycemia, and higher QALYs compared to NPH/Reg insulin.

Weight gain is a common side effect with insulin therapy and could hamper the positive outcomes gained by its anti-hyperglycemic effects [44]. Since the majority of T2DM patients are already overweight/obese, both patients and the physician are concerned that insulinization would result in further gains in body weight. Therefore, this issue is an important determining factor in choosing the type of insulin. In the UKPDS patients taking insulin gained 4 kg more than those treated with diet therapy over 10 years [36]. It has been suggested weight gain can be modified by increasing exercise, restricting calories, and administering metformin concurrently [33, 37]. In the present study, less weight gain was achieved with BIAsp 30 compared with NPH/Reg although, in contrast to our results, in a review of literature weight gain during treatment was not different between BIAsp 30 and biphasic human insulin 30 [45]. However, the benefits of insulin administration outweigh the health risks of increased weight.

Our findings corroborate and complement the IMPROVE™ study which showed addition of BIAsp 30 to treatment protocol of T2DM patients not only substantially improves glycemic control, but also is associated with an enhanced quality of life [17]. Our analysis also revealed that switching from OGLDs to insulin does not increase treatment burden due to injectable insulin neither in patients treated with BIAsp 30 nor in the NPH/Reg arm. In addition, the health status improved in both groups during the study though the change was only statistically significant for the BIAsp 30 patients. Similarly, insulin analogs have been shown to increase patients’ health-related quality of life (HRQoL) compared with human insulin [46, 47]. Patients with improved HRQoL are more likely to adhere to prescribed treatment regimens and insulinization, thereby achieving better glycemic control [48]. Consequently, this may lower the expenses related to poor compliance or delayed adoption of insulin therapy [49]. An observational study in the United States has shown that adherence to insulin therapy increases and incident hypoglycemia decreases when patients are switched from the traditional vial and syringe system to insulin analog pen devices [50]. In another study, Brod et al. found that patients who were treated with BIAsp 30 reported improved treatment satisfaction after 26 weeks and that was large enough to be considered clinically meaningful to the patients [51]. It should also be noted that occurrence of major hypoglycemic episodes and weight gain both have detrimental effects on patients’ HRQoL and enhanced QALYs of the patients receiving BIAsp 30 could in part be accounted for by its superior profile in this regard [52].

### Cost analysis

Increases in health care costs have an important concern for health care policy makers. Many studies have described the economic impact of diabetes on health care systems [53–56]. Considering the findings of two related studies, T2DM and its complications impose a large economic burden on the individual and health care system in Iran [2, 53]. Taking the disease burden and the growing pandemic of diabetes into account, we should select the most cost effective strategies to control the increasing costs. Although insulin analogs are more expensive to the payer than human insulin, insulin analogs may be able to reduce more expensive long-term expenditures such as the costs related to treatment of hypoglycemia or chronic complications of DM [49]. The results of this study demonstrated that treatment with BIAsp 30 was associated with improvements in glucose control and QALY in comparison with treatment with NPH/Reg insulin in patients with T2DM. Treatment with BIAsp 30 was also associated with reduction in total costs rather than NPH/Reg insulin. WHO’s recommendation about threshold of developing countries considers ICER less than triplet of GDP as a cost-effective intervention [28]. Accordingly, treatment of T2DM is categorized as “highly cost effective” if the ICER is less than GDP per capita; “cost-effective” if the cost/QALY was between one to three times of GDP per capita, and “not cost-effective” if it was more than three times of GDP per capita. Our analysis showed that treating poorly controlled patients with BIAsp 30 is likely to be cost-saving and cost-effective in Iran. In parallel to the results of our study, various studies have shown better glycemic control and more tolerability with insulin analogs compared with human insulin in patients with T2DM [57–59]. Subsequently, cost-effectiveness of BIAsp 30 could be attributed to lower incidence of hypoglycemia in short-term and better prevention of DM related complications in long-term [60, 61]. Along the same lines, it has been demonstrated that total annual cost-savings with insulin analogs is 1590 USD per patient, of which 788 USD is hypoglycemia-related cost-savings and 600 USD is due to other DM-related cost-savings [50]. There was not any insulin allergy observed in our patients because all insulins are made using genetic recombinant techniques thus, insulin allergy is uncommon.

## Conclusion

In conclusion, the present randomized, open-label clinical trial revealed that biphasic analog BIAsp 30 is at least as effective as NPH/Reg with respect to glycemic control, and is associated with lower incidence of hypoglycemia episodes and higher QALY. When these gains are translated into cost-effectiveness analysis, it appears that BIAsp 30 is significantly more cost-effective than the NPH/Reg regimen and could better prevent long-term complications of T2DM when prescribed alongside OGLDs.

### Limitations

There were some limitations in this study. We did not calculate cost of hypoglycemia separately, thus the impact of hypoglycemia on total cost was not measurable. In addition, this study was conducted in Iranian population, therefore, the results would be generalizable to other population with caution.

## Abbreviations

BIAsp; 30, Biphasic insulin aspart 30; NPH/Reg, NPH/regular human insulin; T2DM, Type 2 diabetes mellitus; QALY, quality adjusted life year; HbA1c, Glycosylated Hemoglobin; FPG, Fasting plasma glucose; PPG, post-prandial glucose; ADA, American Diabetes Association; OGLDs, oral glucose lowering drugs; PG, Plasma glucose; HPLC, high performance liquid chromatography; HDL, high-density lipoprotein cholesterol; LDL, low density lipoprotein cholesterol; IRR, Iranian Rials; USD, USA dollar; EQ-5D-3 L, European quality of life-5 dimensions,-3 levels; VAS, visual analogue scale; ICER, incremental cost effectiveness ratio; WHO, World Health Organization; GDP, gross domestic product; SD, standard deviation; ANOVA, analysis of variance; HRQoL, health-related quality of life
